# Short-term cortical activation changes associated with postural compensation in swallowing

**DOI:** 10.1007/s00221-024-06928-2

**Published:** 2024-09-25

**Authors:** Kelsey L. Murray, Seng Mun Wong, Erin Kamarunas

**Affiliations:** 1 Communication Sciences and Disorders, James Madison University, Harrisonburg, VA USA; 2 Speech Therapy Department, Singapore General Hospital, Singapore, Singapore

**Keywords:** Functional near-infrared spectroscopy, Hemodynamic response, Dysphagia, Chin tuck, Chin-up, Compensatory strategies

## Abstract

Compensatory strategies used to treat dysphagia, like the chin-down and chin-up positions, are often employed by speech-language pathologists to enhance swallowing safety. However, their effects on cortical neural responses remain unclear. This study aimed to investigate the cortical hemodynamic responses to swallowing across three head positions —chin-down, chin-neutral, and chin-up — using functional near-infrared spectroscopy (fNIRS) in the bilateral precentral and postcentral gyrus regions of interest. Twenty-six healthy adults completed 32 swallows of 5 ml water in each head position. Results revealed short-term cortical activation increases for chin-up swallows compared to both chin-neutral (mean difference = 1.2, SE = 0.18, *p =* .048) and chin-down swallows (mean difference = 0.76, SE = 0.18, *p =* .009). These findings suggest that postural changes during swallowing induce immediate neural adaptations in people without swallowing difficulty. These modifications likely reflect the necessary sensory and neuromuscular adaptations required for safe swallowing in different head positions, with less hyolaryngeal movement needed for a chin-down swallow and more movement needed for a chin-up swallow. While challenging swallow conditions, like the chin-up, may offer promising therapeutic potential, caution is warranted considering the associated safety risk, and further investigation is needed. This study provides insights into the immediate effects of head positions on cortical activity during swallowing and highlights avenues for future research in dysphagia rehabilitation.

## Introduction

Dysphagia is abnormal swallowing function and can be caused by neurophysiological impairment from underlying conditions such as stroke, traumatic brain injury, neurologic disease, oropharyngeal cancer, and gastroesophageal reflux disease (Cho et al. 2015; Epps et al. 2020; Frowen et al. 2020; Lee et al. 2016; Luchesi 2017; Smith et al. 2018). Swallowing involves a brainstem-patterned motor response influenced by cortical and subcortical control and sensory feedback (Jean 2001). Varying central nervous system activation patterns have been demonstrated for automatic versus volitional swallows (Martin et al. 2001), volume changes (Humbert et al. 2009), various head positions (Jestrović et al. 2015, 2016), and sensory modulations (Teismann et al. 2007; Lowell et al. 2008; Humbert and Joel 2012).

To improve swallowing outcomes, speech-language pathologists (SLPs) often implement compensatory strategies to immediately, albeit temporarily, increase the safety of the oropharyngeal swallow. Compensatory strategies can include postural adjustment of the head or neck during eating and drinking, such as the chin tuck (Bulow et al. 2001; Jones et al. 2014; Park et al. 2012; Solazzo 2012). Studies have demonstrated that a chin-down or chin-tuck position during swallowing slows the bolus flow by increasing the vallecular space and can protect the airway by pushing the epiglottis posteriorly to narrow the laryngeal vestibule opening, improving the timing of laryngeal elevation which reduces the risk of aspiration (Balou et al. 2014; Ekberg 1986; Logemann 1983; Logemann et al. 1998; Vose et al. 2014; Welch et al. 1993). A chin tuck position may be specifically helpful in persons with food or liquid present in the pharynx before swallowing onset, usually due to premature spillage from weak lingual control and/or a delay in pharyngeal swallow initiation. Another postural position used in dysphagia treatment is the chin-up position (i.e., head extension), which assists with oral transfer by allowing gravity to shift the bolus toward the pharyngeal cavity. The chin-up is most typically used with patients who have head and neck cancer etiologies and have difficulty with oral transfer (Mittal et al. 2003; Pauloski 2008).

Traditionally postural changes like the chin tuck and chin-up have not been considered to have a long-term role in functional neuromuscular recovery. However, recently postural positions have been shown to result in short-term neural changes. Swallowing in the chin tuck position resulted in short-term changes in electroencephalogram (EEG) *alpha*, *beta*, and *gamma* frequency bands compared to swallowing in a neutral head position, which was attributed to the inhibition of movement during a chin tuck, reallocation of cognitive resources required to complete the task, and differences in muscle recruitment, respectively (Jestrović et al. 2015, 2016). Inhibition of movement may be related to reduced anterior hyoid excursion (Leigh et al. 2015) and reduced laryngeal elevation (Wong et al. 2017), as well as reduced duration of pharyngoesophageal opening (Lee et al. 2020) that can occur during the chin tuck. It could be posited that using a chin tuck is functionally similar to using an ambulatory aid, such as traditional axillary crutches, in that it makes the scale of required movements smaller as a protective mechanism. While ambulatory aids allow for the transfer of weight bearing to the upper extremities, a negative side effect of their use is reduced skeletal muscle activity of the affected lower extremity by as much as 50–65% (Clark et al. 2004). Adaptation to reduced functional load in the lower extremities is evidenced by muscle atrophy, as well as altered muscle architecture and neural activity (de Boer et al. 2008; Deschenes et al. 2002; Sanders et al. 2018; Wall et al. 2013; Wall et al. 2014). Essentially, less movement and load could promote muscle atrophy. There is the potential that using a swallowing compensatory strategy, such as a chin tuck, may result in an altered physiological motor pattern, which could affect sensorimotor neural pathways. While the potential for long-term neuroplastic changes (maladaptive or otherwise) has not been investigated, the principles of neuroplasticity (e.g., use it and improve it) suggest that over-reliance on postural strategies over a prolonged period may affect functional connectivity.

Conversely, the chin-up (head extension) posture increases the distance between the hyoid and the larynx at rest, which increases the size of the laryngeal aditus, and in healthy adults, results in greater laryngeal elevation during swallowing compared to swallowing in a neutral or chin-down position (Wong et al. 2017). These physiological adjustments to swallowing in a chin-up position are likely attributable to greater suprahyoid and infrahyoid contraction during a chin-up position compared to the chin-down position (Sakuma and Kida 2010). This head position also changes the timing of the pharyngeal swallow, with laryngeal vestibule closure occurring earlier in the swallow, while hyoid burst and peak hyoid elevation occur later (Calvo et al. 2017). The authors suggest that using a chin-up position provides a “challenge” to specific pharyngeal swallowing kinematics, which validates the investigation of this maneuver for potential rehabilitative use. Therefore, determining the immediate effect of head position on cortical neural responses is essential, as it can provide invaluable information regarding the potential benefits and drawbacks of these positions for short-term neuroplastic outcomes.

This study aimed to investigate the potential neural effects of chin-down and chin-up positions by examining the cortical hemodynamic responses underlying the adaptation of swallowing movements to varying demands for airway protection across three different head positions: chin-down, chin-neutral, and chin-up. Functional near-infrared spectroscopy (fNIRS) was used to record changes in oxygenated hemoglobin (OxyHb) in the chin-down and chin-up positions compared to chin-neutral. It was hypothesized that swallowing in a chin-up position would increase cortical activation over the primary sensorimotor areas in response to the required increased muscular contraction and movement scaling necessary to achieve laryngeal vestibule closure compared to chin-down and chin-neutral positions.

## Methods

### Participants

The Institutional Review Board approved this study prior to participant recruitment. Participants were recruited from the university. All participants completed a screening in-person, by phone, or online via Qualtrics survey, followed by voluntarily completing the informed consent process. The inclusion criterion was participants with a functional oropharyngeal swallow. Exclusion criteria were any illness or disease process that could affect swallowing including a current or past history of brain injury, neurological disorders, neck injury, head and neck cancer, chronic obstructive pulmonary disease, asthma or other respiratory diseases, psychiatric disorder (excluding medically managed depression), and progressive neurodegenerative disorders (i.e., dementia, Parkinson’s disease, multiple sclerosis, peripheral neuropathy, and amyotrophic lateral sclerosis). Additionally, participants had to have a score of < 13 on the Reflux Symptom Index, indicating they did not experience symptoms of reflux (Abraham and Kahinga 2022; Belafsky et al. 2002).

### Design

This prospective within-subjects experiment compared hemodynamic responses (HDR) to swallowing across three postural positions (chin-down, chin-neutral, chin-up) that are commonly used therapeutically in various populations with dysphagia.

### Instruments and procedure to determine swallow onset

To determine swallow onset time, respiratory and laryngeal movement were digitally recorded. Respiratory induction plethysmography (Respitrace™) was utilized to provide a non-invasive measure of respiratory patterning to identify periods of apnea associated with swallowing. Participants wore elastic bands wrapped around the chest and abdomen to digitize movements associated with respiration. A piezoelectric accelerometer (Kistler, model 8778A500, Amherst, New York, USA) measured the dynamic change of hyolaryngeal elevation associated with pharyngeal swallow initiation. The accelerometer was attached to the skin on the front of the neck at the level of the laryngeal prominence of the thyroid cartilage (i.e., Adam’s apple) using medical tape.

A trained observer marked visualized movements associated with swallowing in the digital file “live” during each session. Additionally, a webcam was positioned to capture video recordings of the participant’s neck movement during the experiment for later review and verification of swallows. These videos were reviewed if there was ambiguity or discrepancy in the digital signal and swallow markings made by the observer. The respiratory plethysmography, piezoelectric accelerometer, live observer verification of swallows, and video recordings were synchronized and digitally recorded using PowerLab 16/35 in Labchart 8 Software (AD Instruments Inc, Sydney, Australia). The respiratory signal was sampled at 1 kHz, and the accelerometer at 4 kHz. The accelerometer signal and observer verification signals were also synchronized with the fNIRS hemodynamic response recordings through auxiliary channels.

The data was analyzed offline to identify the timing of swallow onset for fNIRS analysis, but the signals were not analyzed as outcome measures. The accelerometer signal was rectified for improved offline confirmation of swallow onset, smoothed with triangular (Bartlett) window smoothing (201 samples), and a derivative signal was obtained. Swallow onset was defined as the time point the derivative crossed zero in a positive trajectory during an apneic period, indicating laryngeal movement associated with pharyngeal swallowing onset. Simultaneous visual confirmation of laryngeal and oral movement associated with swallowing from either the trained observer or video confirmation was required.

### Instruments and procedures to determine cortical activity during swallowing

**Fig. 1:**
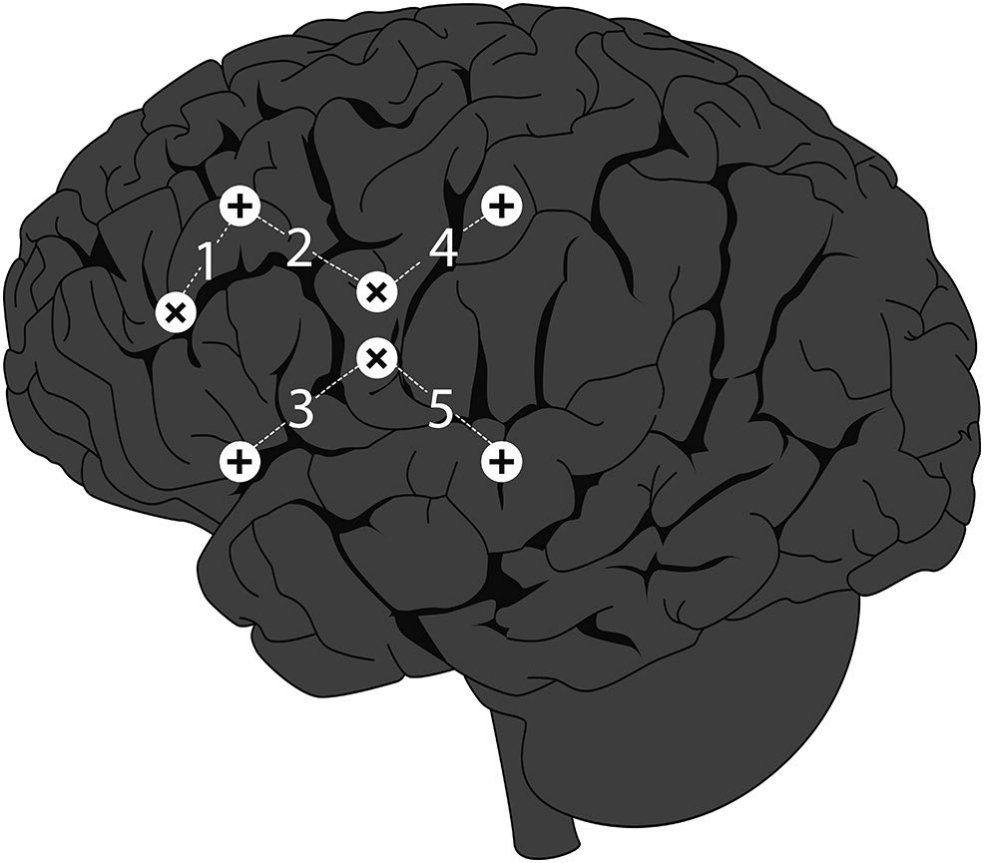
Placement of fNIRS emitters and detectors. Paring 1 is premotor cortex, pairings 2 and 3 are precentral gyrus, and pairings 4 and 5 are postcentral gyrus. X symbol indicates emitters and + symbol indicates detectors

fNIRS was used to measure HDR to swallowing in three head positions (TechEn, Milford, MA, Model CW6). fNIRS provides a non-invasive method of measuring blood oxygenation using optodes that emit near-infrared light (790 and 830 nm), similar to pulse oximetry in concept and practice. Light detectors are placed 3 centimeters from the emitters to absorb reflected light from the emitter. Figure 1 demonstrates the location of the emitters and detector placements to capture HDR in the regions of interest (ROI), including the bilateral premotor cortex, primary motor cortex, and primary sensory cortex. Ten emitter–detector pairs were placed on the scalp, five pairs on each side using a custom created emitter-detector cap. The ROI coordinates (Montreal Neurological Institute, MNI) have been reported in previous protocols (Kamarunas et al. 2022) and are active during swallowing (Lowell et al. 2008; Martin et al. 2004). Brainsight Software v2.0 (Rogue Research, Montreal, Quebec) was used to guide emitters and detectors’ placement from a standard sex-matched MRI. The fNIRS sampling rate was 50 Hz. Following the initial placement of each ROI, repeated measurements were conducted and verified to be within a 5 mm proximity of the target ROI.

Participants were seated in an upright exam chair with arm and head rests and their feet comfortably flat on a footrest. Appropriate head angles for each of the three head positions were then defined for each participant. The participant wore glasses (without lenses) with a laser pointer attached to it just above the participant’s left ear to project a beam onto the wall approximately 8 feet in front of the participant. The participant was requested to hold their head in a comfortable neutral position and, holding this position, a small target was placed on the opposite wall so that the laser shone on the target when the participant was in a neutral head position. This allowed the participant to consistently place the laser light in this target to maintain the same neutral head position over time. From the neutral position, the participant was then guided to tilt their head up to 15° above neutral and then 15° below neutral using a digital goniometer. Circular targets for both the up and down positions were also placed on the wall in front of the participant to guide the transition and maintenance of head positions during the experiment. Participants were instructed to keep the laser light within the designated target while maintaining each head position.

Tygon^®^ B-44-3 beverage tubing (3/32” ID x 5/32” OD x 1/32” wall) was attached to the participant’s cheek for oral delivery of distilled room temperature water boluses via a water pump (MasterFlex^®^ L/S, Model 77200-52, Cole-Palmer). Every 35 s, 5 ml of water was delivered across three seconds with a flow rate of 100 ml/min. A small microphone was used to record the sound of the water pump, which was synchronized with the digitized physiological signals, so offline analysis was aided by bolus delivery information.

**Fig. 2:**
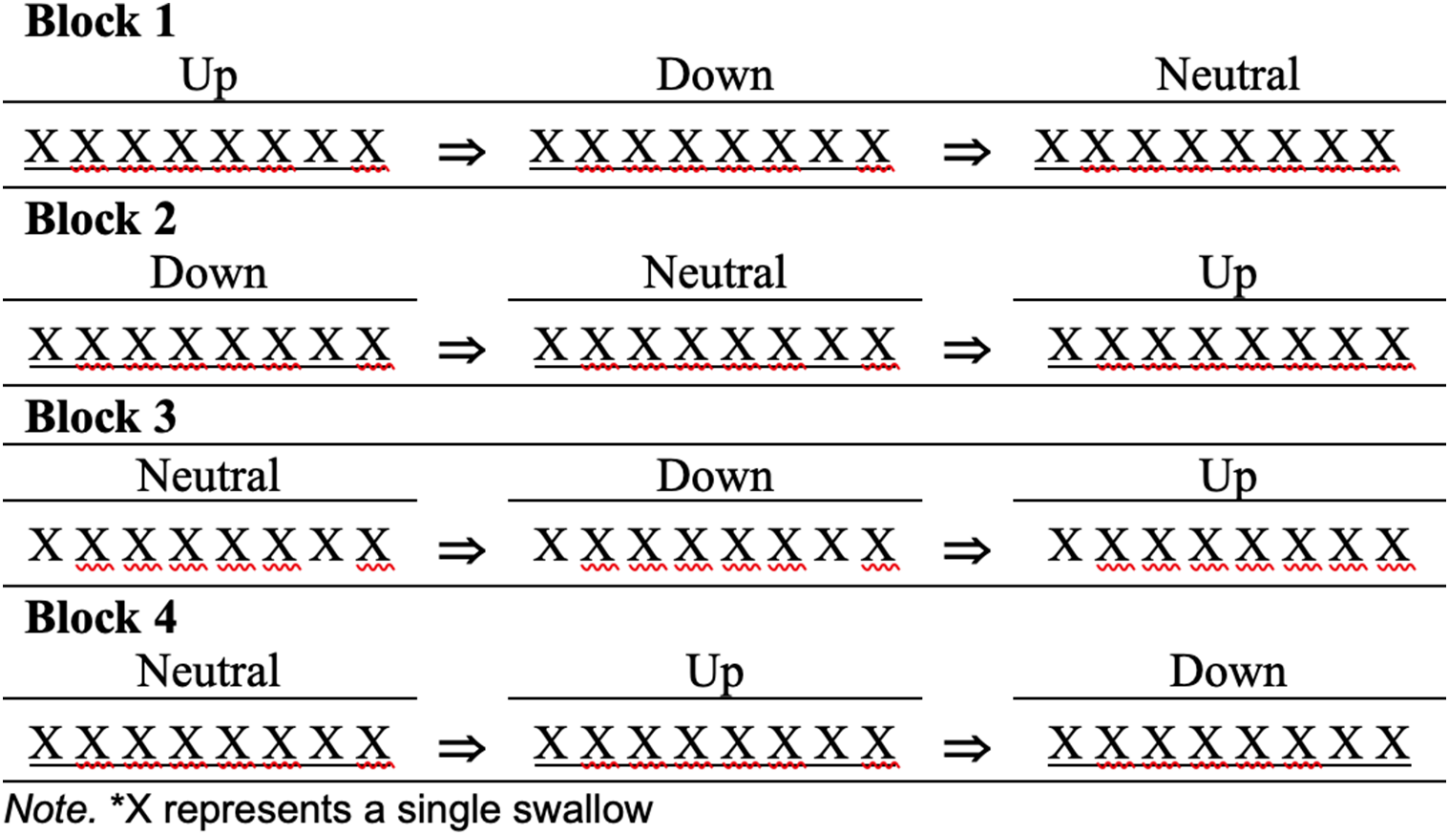
Example of randomized order of blocks divided into three head positions for a single participant. Each participant had a randomized order of head positions within each of the four blocks

FNIRS recordings occurred in four 16-minute blocks. During blocks, the participant maintained each head position approximately 5–6 min, swallowing every 38 s, so that 8 consecutive swallows were completed in each head position. The order of head positions were randomized for each block (Fig. 2). Changes in head position were visually cued with an up, down, or neutral arrow. Participants were instructed use slow movements to change head positions to avoid motion artifacts in the fNIRS signal. They were generally instructed to swallow when they received a water bolus but were not visually or verbally cued to swallow. They were given 3–4 min to rest between each block. Each participant completed eight consecutive swallows in each head position across four blocks for a total of 32 swallows in each head position (Fig. 2).

Homer2 software (Boas et al. 2012) in Matlab 2013 (The MathWorks Inc., Natick, MA) was utilized to process fNIRS data offline. fNIRS data from Homer2 were time synchronized with onset swallows from Labchart 8 by a shared auxiliary signal. Raw wavelengths were converted to optical density values. A bandpass filter at 0.01–0.5 Hz removed physiological signals (Mayer’s waves, respiratory, cardiac). Optical density was converted to hemoglobin concentrations with the application of the modified Beer-Lambert law. Motion correction was completed using adaptive short-separation filtering followed by a correlation-based signal improvement filter to remove any artificial motion (e.g., temporalis) (Cui et al. 2010). Event-related averages of oxygenated hemoglobin (OxyHb) from − 10-s to 20-s from swallow onset were exported from HOMER2 for analysis, in two ways: In the first analysis, all swallows were exported by condition; in the second analysis, the first two swallows in condition blocks and the last two swallows in condition blocks were exported to compare activation habituation over the series of swallows in a block.

Baseline data was defined as −10-s to 0-s prior to swallow onset. OxyHb signals were normalized to baseline cortical activity by subtracting the mean baseline value from each channel individually. Baseline swallow means were verified to be consistent across time within each block (i.e., beginning of block/end of block) for all head positions (*p* > .05).

The median value from the 5-s to 20-s window from swallow onset (time 0) for each condition was calculated across all channels. This window was chosen because the cerebral blood flow response to swallowing typically peaks 4–6 s after the neural response and starts to return to baseline at 20–30 s (Kamarunas et al. 2018). Median values from the channels in each hemisphere were separately averaged.

### Analysis

Statistical analysis was completed in R statistical software (v 4.4.0; R Core Team, 2024, Vienna Austria). A backwards regression procedure using the ‘stats’ package (v 4.4.0; R Core Team, 2024, Vienna Austria) was run to determine which variables (head position, timing (i.e., first or last swallows in a block), and hemisphere side) would change OxyHb levels and then followed by a post-hoc analysis. The ‘lme4’ and ‘lmertest’ packages (v1.1.35.3; Bates et al. 2015; v3.1.3; Kuznetsova et al. 2017) were used to construct a multiple linear regression model and determine the best-fitting model for the change OxyHb. Alpha level was set to .05.

## Results

**Fig. 3:**
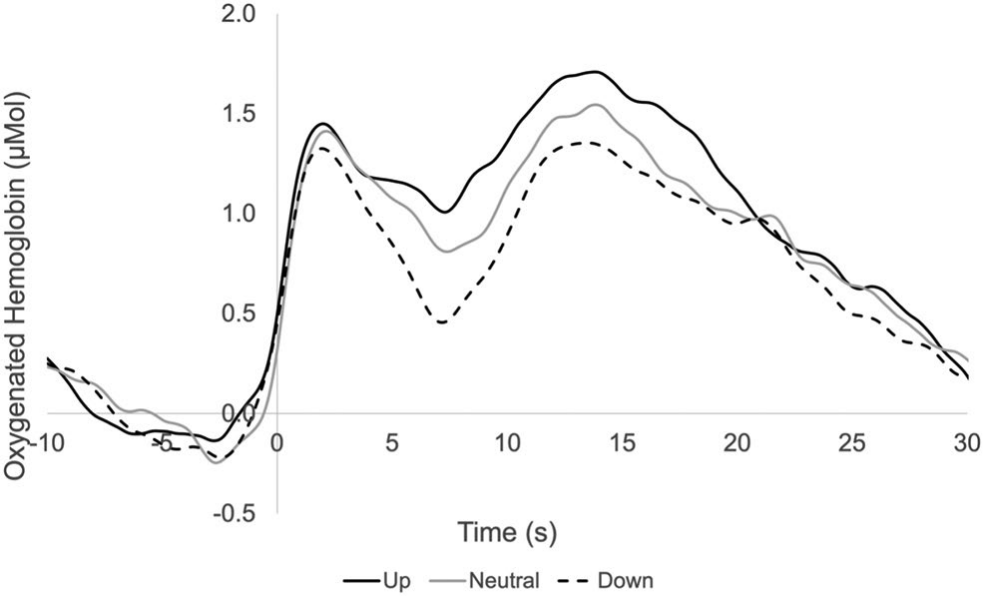
Mean OxyHb signal for the three head positions from 10 s before swallow onset to 30 s after, averaged across all participants. Time at 0 s represents swallow onset

Twenty-six healthy adults without swallowing difficulties were included (female = 24, mean: 23 years, range 20–36 years). Table 1 provides the descriptive statistics for OxyHb means for each head position by hemisphere across time. Figure 3 depicts the OxyHb means over time for all three head positions.

**Table 1:** Hemodynamic response by head position, hemisphere, and timing

fNIRS descriptive statistics: mean OxyHb (SD), *n* = 26						
Head position	Left hemisphere			Right hemisphere		
	First in block	Last in block	All swallows across block	First in block	Last in block	All swallows across block
Down	0.50 (0.91)	1.05 (1.41)	0.59 (0.84)	0.47 (1.00)	0.58 (0.98)	0.93 (1.04)
Neutral*	0.52 (1.21)	1.28 (1.58)	0.68 (0.96)	0.64 (1.27)	0.60 (1.34)	1.2 (1.46)
Up †	1.46 (3.3)	1.25 (1.78)	0.95 (1.29)	1.39 (2.23)	0.72 (1.22)	1.39 (1.45)

*Note*: Units are µM, *Comparison to chin-down *p* < .05; †Comparison to chin-down *p <* .01

### Linear modeling

Backward elimination regression analysis was conducted to identify the best-fitting model for the variables that influence OxyHb during a swallow. For the OxyHb outcome variables, six variables were included (three independent variables and three interactions). The most complex model included: head positions, timing within a head position block (first/last), hemisphere (left/right), and interactions between these variables. The model also included a random effect to control for participant, since this is within participant data. Only the output from the best-fitting model is reported.

The backward procedure determined that the best-fitting model for change in OxyHB included one independent variable: head position (Estimate = 0.56, SE. = 0.19, *p* = .003). Therefore, the independent factors that identified a significant change in the OxyHB included head position. A *post-hoc Tukey* test was completed using the package ‘emmeans’ (Lenth 2024) to explore differences between head positions. The pairwise comparison revealed significant changes in OxyHb between head position chin-down and chin-up (mean difference = .76, SE = .18, *p =* .009) and chin-neutral and chin-up (mean difference = 1.2, SE = .18, *p* = .048) (Table 2).

**Table 2:** Post-hoc of head positions for OxyHb changes

Head position	Mean differences	SE	*p* value
Down vs. neutral	0.65	0.18	0.82
Down vs. up	0.76	0.18	0.009**
Neutral vs. up	1.2	0.18	0.048*

*Note*. * *p* < .05, ** p < .01

## Discussion

This study examined the differences in cortical hemodynamic responses to swallowing across three different head positions. The results indicated short-term cortical activation (OxyHB) increases associated with the degree of head extension during swallowing, with the chin-down position resulting in the least activation and the chin-up resulting in the most. These cortical activation changes may reflect the neural response to the increased hyolaryngeal movement scaling required for adequate laryngeal closure as the degree of head extension increases (Wong et al. 2017). The reduced activation observed during the chin-down head position may reflect the reduced movement required since the anatomical structures were likely closer in proximity (Welch et al. 1993). There were no effects of hemispheric laterality or habituation of cortical activity over the 5–6 min they maintained each head position.

The chin-tuck has been widely studied and has been shown to improve airway protection by shifting the anterior pharyngeal structures more posteriorly to increase the vallecular space and narrow the laryngeal opening (Leigh et al. 2015; Welch et al. 1993). The chin-tuck also leads to earlier laryngeal vestibule closure and later opening (Young et al. 2015). Its effectiveness at reducing aspiration is reportedly low (25–34%) (Ko et al. 2021; Logemann et al. 2008; Nagaya et al. 2004), with reduced effectiveness if stasis is present in the pyriform sinuses (Shanahan et al. 1993) and if dysphagia is severe (Saconato et al. 2016). The chin-tuck may also limit hyolaryngeal range of motion during swallowing due to reduced scaling (Leigh et al. 2015; Wong et al. 2017). In other words, when the structures are brought closer together during a chin-tuck, the necessary hyolaryngeal range of motion to achieve laryngeal closure are now smaller so smaller movements are now used. For patients reliant on a chin-tuck for swallowing safety, it is unknown if consistently limiting swallow hyolaryngeal range has any long-term consequences for swallowing recovery, like the use of ambulatory aids on lower extremity function (de Boer et al. 2008; Deschenes et al. 2002; Sanders et al. 2018; Wall et al. 2013; Wall et al. 2014). Our data suggests that there is a short-term reduction in cortical sensorimotor activation when swallowing in a chin-down compared to a chin-neutral position, but whether there are long-term implications for this is unknown.

The immediate effects of the chin-tuck position on functional neural connectivity have previously been studied. Swallowing in a chin-tuck position resulted in increased functional connectivity compared to a neutral head position, which, for specific frequency bands, was attributed to the chin-tuck having decreased or different muscular recruitment (Jestrović et al. 2015, 2016). While it is difficult to compare the outcomes of this fNIRS study to similar studies done with EEG, it does appear that there may be some differences in interpretation. Our results can be interpreted as the chin-down position resulting in decreased activation in the premotor and primary sensorimotor cortical areas, possibly also related to decreased movement associated with the swallow in that position. Likely, these are not mutually exclusive conclusions and reflect the differences in interpretability of the outcome measures.

The chin-up posture optimizes gravitational bolus flow, especially through the oral cavity, but also increases the size of the laryngeal opening and requires greater hyolaryngeal movement to achieve laryngeal closure (Wong et al. 2017). Supra- and infrahyoid contraction amplitude and duration were greater in a chin-up position compared to a chin-down position (Sakuma and Kida 2010). These increases were significantly associated with perceived difficulty in swallowing (Sakuma and Kida 2010). It is likely that the increases in cortical activation for chin-up swallows in this study reflect the neuromuscular response required to achieve a safe swallow in a more complicated position, given the mechanical differences between the two positions. While a mechanistically challenging and perceptually difficult swallow strategy is presumably not ideal for all patients with dysphagia, it is possible that a postural position that promotes increased muscle contraction and cortical activity may be a useful rehabilitation tool for adding intensity load to swallowing exercises. This is especially important as many strength-based swallowing exercises do not easily allow for systematically and quantifiably progressing physiological load over the course of therapy (Burkhead et al. 2007). Additionally, a recent study identified an increase in cortical activation during effortful swallowing compared to normal swallowing, which aligns with this study in terms of challenging the neuromuscular response (Chua and Chan 2024). Therefore, a patient could use a chin-up position starting with a small degree of head extension (e.g., chin up 10°) and couple this position with swallow-specific rehabilitation exercises, such as the Mendelsohn maneuver or effortful swallow, to add an additional “load” or challenge to the exercise. As with any swallowing exercise with patients with dysphagia, special consideration would have to be taken for the safety of swallowing a bolus in this position, which may include carefully choosing bolus consistency and size, or whether a bolus is used at all. As treatment progresses, the exercise load could be increased incrementally (e.g., adding 5° of head extension). Certainly, this is a conceptually interesting avenue of research that needs further investigation before recommendations can be made.

## Future direction and limitations

This study had a limited sample size (*n* = 26) and age range (20–36 years). Additionally, it included only non-dysphagic participants. These factors limit interpretation and generalization to the older population and those more medically fragile. Additionally, a chin-down position in this study was defined as 15° below a comfortably neutral position. However, 17.5° of neck flexion has been previously recommended as the minimum to achieve a benefit from the chin-tuck (Ra et al. 2014). Although the difference is minimal, it is possible that the difference of 2.5° in flexion and extension may have altered our results.

This study only examined the immediate hemodynamic effects of swallowing in different head positions. Future research may consider investigating the long-term effects of postural changes, especially the chin-tuck when used as a safe swallow strategy on long-term motor recovery or maintenance. The findings from this and other related studies suggest that providing patients who are undergoing swallowing rehabilitation with a “challenging” swallowing condition through postural positioning may promote increased neuromuscular involvement. Stage 0 and 1 clinical trials are needed before the safety and effectiveness of exercise loading with head position can be determined.

## Conclusion

Swallowing in different head positions causes short-term changes to the sensorimotor cortical activation in people without swallowing difficulty and likely reflects the neuromuscular changes required to swallow safely in each position. Whether these short-term changes in HDR have the potential to influence long-term neural plasticity is yet unknown. While this may hint at a promising future treatment modality for more challenging swallowing positions, such as the chin-up, long-term effects may be less promising for movement-inhibiting positions, such as the chin tuck. More research is needed before conclusions about long-term effects can be made.

## Data Availability

Data generated or analyzed during this study are not publicly available due to confidentiality agreement with research collaborators.
